# 

**Published:** 1906-06-16

**Authors:** 


CONTENTS.

**Military Sanitation** - 187
**Local Anæsthesia in General Surgery** - 188
**Annotations** - 189
**Medical Opinion and Movement** - 190
**Hospital Clinics—**
The Present Position of Radiation in Treatment - 191
**Practical Notes on Diagnosis and Treatment** - 195
**Practical Notes on Remedies** - 196
**The Book World of Medicine and Science** - 197
**Medical Appointments and Vacancies** - 197
**Hospital Administration—**
Current Hospital Topics - 198
Aids to Poor Sufferers - 199
Hospital Meetings - 200
House and Office Heating - 201
**Editor's Letter-Box** - 202

*NURSING SECTION.*

**Notes on News from the Nursing World—** Royalty and the Nursing of Europeans in India—The Government and State Registration—The Queen's Nurse and the Sick Child: Official Contradiction—"Mental Science" Nurses—A Children's Nurses' Column—Guardians and Salaries—The Advantage of Written Agreements—Sick Leave and Salary—An East-End Function—The Pension Fund and Newcastle Nurses—Social Amenities at the Antipodes—Californian Nurses in Need—Peckham Nursing Association—A Second Sister for Oberammergau—Nurses' Union—A Prosperous Provincial Association—Short Items - 155-158
**The Nursing Outlook** - 1'9
**The Care and Nursing of the Insane** - 160
**The Nurse's Clinic** - 161
**The Royal National Pension Fund for Nurses** - 162
**Are Nurses Underpaid ?** - 165
**Laying the Foundation-Stone of the Nurses' Home at University College Hosp't 1l** - 166
**Appointments** - 167
**Everybody's Opinion** - 167
**Notes and Queries** - 168
**For Reading to the Sick** - 168

